# Medical Education—Its Tendencies

**Published:** 1860-11

**Authors:** 


### MEDICAL EDUCATION—ITS TENDENCIES.

The opening of the medical schools inaugurates the medical session of the year. No annual event, properly considered, is of equal importance to the republic of medicine. Yet we fear that it too often passes unheeded by our profession, simply because its significance is not appreciated. Let us consider its bearing upon the future of American medicine. The four or five thousands of students who are now gathering in the schools throughout the country, are the recruits who are to replenish and swell the ranks of that army of practitioners which now numbers, in this country not far from forty thousand. Is it of little consequence that these recruits are qualified by education, habits, and moral training for the peculiar service of the physician! They are to be our brethren, our equals, and in the progress of events they are to be the exponents of the character of our profession, and give it rank in the popular regard. If they are thoroughly qualified by previous education, and bring to the investigation of the abstruse science of medicine, minds well disciplined to patient study and accurate research, then will they become masters in its various departments, and in subsequent life will sustain its reputation as a learned profession. If in addition to educational qualifications, they have correct morals, and sensibilities keenly alive to the sufferings of their fellows, then will they confirm its reputation as the most humane profession. But if the majority of those who are now about to enter our ranks have but a limited education, dissolute and profligate habits, and are seeking personal aggrandizement as the end and aim of life, then they will degrade the profession to which they belong, in the estimation of all whose opinion is entitled to respect and consideration. Could we determine the character of the recruits that are to-day admitted to the ranks of the army, we could with certainty foretell the value of that army, when the struggle of the conflict comes. We need scarcely add that if we judge from the past, many who now enter upon their medical studies have no proper qualifications. We could wish that it were not so; that those who stand at the threshold of the temple as its guardians, would carefully scan the applicants for admission, and turn away to more congenial pursuits, the ignorant, the immoral, the unworthy. Every association of men, for whatever purpose, guards vigilantly the door through which accession is gained to its ranks. The wisest and most trustworthy are stationed at the portals to examine each candidate that no improper person may become a member of its select body, and change the peculiarity of its original organization. But the ancient and honorable profession of medicine gives little heed, in this country at least, to the character and trustworthiness of those who guard the portals of its temples. Unconcerned it witnesses the annual influx of members, and sees the most unworthy too often elevated to the privileges and honors of its order without a remonstrance. It is true that hitherto the profession, as a body, has lacked the organization, and consequently the power, to protect itself from these degrading associations. The field of legitimate medicine, like a wide domain imperfectly hedged, is guarded by mercenary sentinels, and thousands, unqualified, annually purchase admission, and with the most meritorious, garner its rich fruits. But a better day is dawning upon American medicine, and a brighter era will ere long occur in its history. The profession at large has an organization which is already sufficiently powerful, were its forces but properly directed, to protect its own domain from further incursions. Through the medium of the American Medical Association, it can erect such defences as it chooses, and dictate, authoritatively, who may, and who shall not, be admitted to its highest privileges. That it cannot compel the educating bodies, as by legal force, to scan more closely the preliminary qualifications of students, and indicate the standard of educational qualifications of graduates, is very true; but it can by suitable organization establish its *own* standard of education, have its *own* examining body, and confer its *own* degrees. The exigencies of the times demand this of the American Medical Association; the honor, dignity, and character of American medicine are approaching a crisis which this body can avert. We may not now indicate the precise steps by which this great reform is to be accomplished, but that the initiatory step must soon be taken, and the work resolutely prosecuted to its consummation, no one who has at heart the honor of our profession can for a moment doubt. In the collection of medical schools which it was our privilege to present in the student's number of the *Medical Times*, we have, we think, lain a foundation for rational speculation in regard to medical education in the United States. It not only affords the opportunity, much needed, of learning the advantages which the schools in different sections of the country offer to students, but what is of more consequence, we there learn the value which each school attaches to its diploma. This valuation indicates their standard of medical education. It is not our intention at this time, to enter upon that critical examination of the subject of medical education, to which this collection invites us, but simply to offer some general conclusions which are apparent on a superficial examination. What will, perhaps, prove to the mass of readers the most marked difference in our medical schools, has a sectional bearing, viz: between the Northern and Southern schools. It will be noticed that the fees in the Southern schools are uniformly high, those most recently established having a scale as high as the largest and most favored schools of the North. Among the Northern schools the scale of fees varies from the lowest of the Southern schools, to the price of the parchment for a diploma. If the scale of fees indicates anything as regards the estimate of the school of its educational advantages, and the value of a thorough medical education, this exhibition of figures shows a vastly higher appreciation of a medical education at the South than at the North. The next most striking feature in the schools is the almost universal interest now manifested in clinical instruction. This is, indeed, the most hopeful sign of the times. Heretofore the importance which the schools attached to clinical advantages depended entirely upon the facilities which their particular location happened to afford. The school so unfortunate as to have a situation distant from any hospital or infirmary, loudly decried clinical instruction, and many will remember that a venerable professor went so far a few years ago, as to regard it as absolutely injurious to the student. Schools situated in our lake and seaport towns, saw their advantage, and vaunted their facilities for clinical instruction, and, not unfrequently published in their annual circulars a list of all the medical institutions of the town, many of which were not even open to a transient visitor. Although clinical instruction, as given in our colleges and hospitals lacks system, and is as inefficient as it well can be, still we attach to it so much importance, that we regard this evident desire on the part of the schools to afford such advantages to their pupils as in the highest degree encouraging. Again, it will be noticed, that nearly all of our most flourishing schools have large Faculties, and lengthened courses of instruction, several extending their terms to five months. This fact is worthy of notice, as it is due to the direct influence of the American Medical Association. In concluding these desultory remarks, which the opening of the medical session has suggested, we may add that a careful observation of the history of our educational bodies for the last few years, reveals certain inevitable tendencies which afford reliable data from which to cast the horoscope of the medical schools of this country. Clinical instruction is to become the *sine qua non* in a course of medical education, and hence those colleges located in populous towns which abound in public medical charities, will make the strongest appeal to students, and gain the largest classes. Those cities, again, which offer to the schools the largest ad vantages for hospital practice, will become inevitably the centers of medical education. Nor is it difficult, in the light of the above facts, to indicate the cities which are to be crowned with this proud distinction. That different sections of our wide extended republic must have their own schools of medicine, in which the differences of diseases dependent upon climate are to be especially taught, is evident. The North must have her own schools, and the South and West must have theirs. Already the Pacific coast constitutes a fourth climatic division which must have its schools. The great emporia of these grand divisions of the country must become the centres alike of commerce and education.—*American Medical Times.*

